# Identification of key genes in chickpea transcriptomics and the development of ChickpeaOmicsR as a comprehensive resource to advance breeding and genomic studies

**DOI:** 10.3389/fbinf.2026.1727493

**Published:** 2026-03-10

**Authors:** Alsamman M. Alsamman, Khaled H. Mousa, Asmaa E. Abd El-Hak, Doaa A. Korkar, Anas M. Saedwi, Sandy Khaled, Al-Sayed Al-Soudy, Achraf El Allali, Zakaria Kehel, Morad M. Mokhtar

**Affiliations:** 1 International Center for Agricultural Research in the Dry Areas (ICARDA), Giza, Egypt; 2 Agricultural Genetic Engineering Research Institute (AGERI), Agricultural Research Center (ARC), Giza, Egypt; 3 College of Chemical Sciences and Engineering (CCSE), Chemical and Biochemical Sciences (CBS), Mohammed VI Polytechnic University (UM6P), Ben Guerir, Morocco; 4 Bioinformatics Laboratory, College of Computing, Mohammed VI Polytechnic University, Ben Guerir, Morocco; 5 Genbank, International Center for Agricultural Research In the Dry Areas, Rabat, Morocco

**Keywords:** Cicer arietinum L., differentially expressed genes (DEGs), GWAS, R programming language, RNA-seq

## Abstract

**Introduction:**

Chickpea (*Cicer arietinum* L.) is a key legume crop and a major source of dietary protein in developing countries, yet its productivity is constrained by multiple biotic and abiotic stresses. Advances in RNA-seq and whole-genome sequencing enable detailed exploration of stress-responsive gene expression, but existing resources lack integrated, user-friendly tools for multi-omics analysis in chickpea.

**Methods:**

This study analyzed transcriptomic responses to six stress conditions—drought, heat, cold, salinity, Fusarium infection, and developmental stages—using publicly available RNA-seq datasets. We identified differentially expressed genes (DEGs), enriched gene ontology (GO) terms, and protein–protein interaction (PPI) networks. Critically, we developed ChickpeaOmicsR, the first comprehensive R package that automates the integration of transcriptomic, genomic, and proteomic data and standardizes fragmented chickpea gene nomenclature; enables breeders without bioinformatics expertise to perform complex analyses (e.g., DEG identification, PPI visualization, GWAS integration) in minutes; and provides pre-validated datasets and analytical workflows unavailable in existing tools.

**Results:**

Each stress triggered distinct molecular pathways. Drought and heat stress affected cell wall organization and defense responses, while cold stress influenced circadian rhythm genes. Fusarium stress involved pathways related to innate immunity and secondary metabolism. Developmental stages showed the highest transcriptome variability among the conditions tested.

**Discussion:**

The development of ChickpeaOmicsR addresses critical gaps in chickpea research infrastructure. By providing an integrated and accessible tool that enables complex analyses for breeders without bioinformatics expertise, it accelerates the discovery of stress-resilient genes and the development of improved chickpea varieties.

## 1 Introduction

Chickpea (*Cicer arietinum* L.) is one of the important grain legumes that are grown and consumed worldwide (Ullah et al., 2020). It is a valuable crop that provides nutritious food for the growing world population, and its importance will increase to help address the challenges of climate change (Merga and Haji, 2019). It is a better source of protein, fat, fibre, and carbohydrates than other legumes such as pigeon pea, black Gram, and green Gram (Kaur and Singh, 2007). The world’s population is rapidly increasing and is expected to reach approximately 10.3 billion by the 2100. More than 75% of the world’s population is expected to live in Africa and Asia, which also contain the majority of developing nations, leading to increased risks of food insecurity (Singh et al., 2019). Chickpea is playing a key role in filling the protein gap in developing countries, particularly in Asia and Africa, where energy and protein are obtained mainly from cereals and legumes (Tassoni et al., 2020). Therefore, providing food for the world’s more than seven billion people is one of today’s most pressing challenges (McMichael and Schneider, 2011).

Globally, chickpea productivity is adversely affected by several biotic and abiotic factors (Deokar and Tar’An, 2016). In response, numerous techniques and strategies have been developed to identify genes involved in stress responses (Jain and Chattopadhyay, 2010). Among these, high-throughput RNA sequencing (RNA-seq) technology has become a standard method for measuring RNA expression levels. RNA-seq allows for the detailed identification of gene isoforms, nucleotide variations, and post-transcriptional modifications. A key goal of RNA-seq is to identify differentially expressed genes under various conditions (Moenga et al., 2020). Additionally, genome-wide association studies (GWAS) have emerged as powerful statistical tools for identifying potential genomic regions that could be utilized in breeding programs (Alsamman et al., 2024). GWAS provides valuable insights for marker-assisted selection and helps researchers understand the genetic variation controlling key agronomic traits, ultimately enhancing breeding efficiency (Raman et al., 2022).

Chickpea research faces significant challenges in keeping pace with the vast volume of genomic and genetic information generated daily. Imagine being a breeder with a limited background in genomics, transcriptomics, and genome-wide association studies. You would need to navigate multiple analysis techniques, sourcing data from various sources. Additionally, chickpea genomics suffers from inconsistencies in gene nomenclature and different genome reference standards. These complications restrict progress in chickpea research, forcing breeders to spend countless hours gathering data instead of focusing on developing sustainable and improved genotypes.

Current bioinformatics resources, including general R packages (e.g., DESeq2) and specialized databases (e.g., LegumeIP Dai et al. (2021)), do not effectively meet the specific needs of chickpea research. These tools require extensive manual data curation, lack species-specific analytical processes, and do not provide an integrated platform for breeding applications. To address these issues, we created ChickpeaOmicsR—the first specialized R package that integrates multi-omics data (RNA-seq, GWAS, PPI networks) within a unified and reproducible framework. It automates complex analyses (such as DEG identification, GO enrichment, and trait-gene association) through streamlined computational pipelines. It also standardizes chickpea gene annotations using the current reference genome, and offers pre-validated, ready-to-use datasets to avoid data acquisition delays.

Building on this foundation, our study aims to identify the genetic mechanisms underlying chickpea’s responses to diverse stressors. By analyzing transcriptomic profiles to identify genes associated with drought, heat, cold, salinity, Fusarium wilt infection, and various developmental stages, we identified key genes involved in stress tolerance and growth, including those responsive in multiple contexts. This provides critical insights into biological pathways and protein interaction networks, advancing both the fundamental understanding of chickpea genetics and the development of stress tolerance strategies. Additionally, we have developed a comprehensive, user-friendly R package designed as a centralized resource for the chickpea research community. This package serves as a flexible database, providing accessible tools for data exploration and multidisciplinary analyses. By integrating diverse resources and analytical techniques, it streamlines research workflows and supports both breeders and geneticists in advancing chickpea research.

## 2 Materials and methods

**FIGURE 1:**
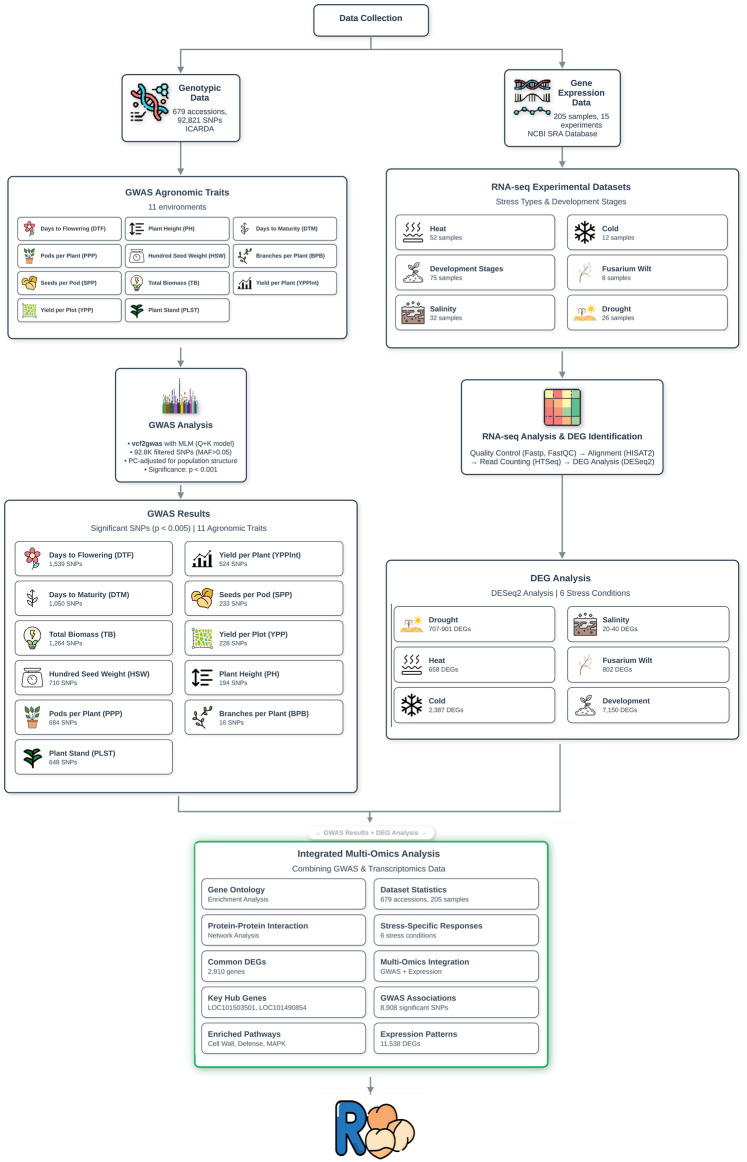
Overview of the ChickpeaOmicsR package, highlighting its capabilities in integrating genetic data for chickpea research. The illustration showcases gene expression and genetic information for genes involved in the flowering-related biological pathway, demonstrating how the package facilitates visualization of key molecular interactions and pathways.

In order to support the ChickpeaOmicsR package, a comprehensive multi-omics analysis was performed, integrating different genomic, transcriptomic, and phenotypic datasets linked to chickpea breeding and molecular research. The major challenge in this effort was ensuring not only rigorous quality control and appropriate analytical methods for each data type but also harmonizing and aligning these heterogeneous datasets to provide a unified, complementary perspective. This integration enables a grand overview of how various biotic and abiotic stresses influence chickpea biology, and how it could be related to economic agronomic traits (Figure 1).

### 2.1 Transcriptome data collection

The genomic data of chickpea were obtained from the NCBI database (https://www.ncbi.nlm.nih.gov). The size of the genome assembly is 530.8 Mb with a coverage of 120.0x. To identify differentially expressed genes, chickpea transcriptome data were acquired under various stress conditions from the Sequence Read Archive (SRA) database (https://www.ncbi.nlm.nih.gov/sra/). For heat stress, PRJNA748749 contains 52 samples, including 28 control samples and 24 stress samples. For the developmental stages, three datasets are listed in this category: PRJNA413872 with 27 samples, PRJNA316845 with 24 samples, and PRJNA316844 with 24 samples. Cold stress includes three datasets: PRJNA903665 with 4 samples, PRJNA905065 with 4 samples, and PRJNA232700 with 4 samples. For salinity stress, the datasets are PRJNA288473 (16 samples), PRJNA232700 (8 samples), and PRJNA579008 (8 samples). Fusarium wilt is represented by two datasets, PRJNA891950 and PRJNA246444, each comprising 4 samples. Drought stress includes three datasets: PRJNA335939 with 4 samples, PRJNA288321 with 14 samples, and PRJNA396819 with 8 samples. All these details are presented in Table 1.

**TABLE 1:** Overview of RNA-seq experimental datasets used for gene expression analysis in chickpea, retrieved from the NCBI SRA database. The table summarizes stress types or developmental stages, corresponding SRA project accessions, experimental comparisons, total number of samples, and biological replicates per condition. Note 1: Seedling vs. Germination vs. Vegetative vs. Reproductive vs. Senescence.

Types of stress	Datasets	Experimental conditions	No. of samples	No. of replicates
Heat	PRJNA748749	Control vs. Heat Stress	52	28 vs. 24
Development stages	PRJNA413872	Five stages (see Note 1)	27	2 vs. 3 vs. 4 vs. 9 vs.9
	PRJNA316845	Seedling vs. Mature	24	21 vs. 3
	PRJNA316844	Seedling vs. Mature	24	21 vs. 3
Salinity	PRJNA288473	Control vs. Salinity Stress	16	8 vs. 8
	PRJNA232700	Control vs. Salinity Stress	8	4 vs. 4
	PRJNA579008	Control vs. Salinity Stress	8	4 vs. 4
Cold	PRJNA903665	Control vs. Cold Stress	4	2 vs. 2
	PRJNA905065	Control vs. Cold Stress	4	2 vs. 2
	PRJNA232700	Control vs. Cold Stress	4	2 vs. 2
Fusarium	PRJNA891950	Control vs. Fusarium	4	2 vs. 2
	PRJNA246444	Control vs. Fusarium	4	2 vs. 2
Drought	PRJNA335939	Control vs. Drought Stress	4	2 vs. 2
	PRJNA288321	Control vs. Drought Stress	14	7 vs. 7
	PRJNA396819	Control vs. Drought Stress	8	4 vs. 4

### 2.2 Genomic data collection

The International Center for Agricultural Research in Dry Areas (ICARDA) is one of the largest gene banks and genetic resource centers for chickpea in Africa and Asia. In previous research (Khan et al., 2024), ICARDA contributed 679 chickpea accessions. These accessions were genotyped using next-generation sequencing technology, yielding approximately 4 million SNPs across the genome. After filtering with TASSEL (Bradbury et al., 2007), 92,821 SNPs were retrieved with a minor allele frequency greater than 0.05 and SNP/sample missing data less than 0.1. These genotypes were evaluated for several agronomic traits, including days to flowering (DTF), plant height (PH), days to maturity (DTM), pods per plant (PPP), hundred seed weight (HSW), branches per plant (BPP), seeds per pod (SPP), total biomass (TB), yield per plant (YPPlnt), yield per plot (YPP), and plant stand (PLST) across 11 environments over 2 years.

### 2.3 Chickpea transcriptome data analysis

#### 2.3.1 RNA sequencing analysis

To explore transcript expression and identify stress-responsive genes, RNA-seq analysis was performed on 15 experiments (Table 1). The raw FASTQ files were processed by removing adapters and quality filtering using fastp (Chen et al., 2018) and fastq-quality-filter (Fu and Dong, 2018) with the following strict parameters: a minimum Phred score of 20, a minimum read length of 45 bp, a sliding window size of 4 bp, and a mean quality of 20 within the window. Subsequently, the quality of the processed reads was assessed using FastQC (Li et al., 2015). The clean reads were then aligned with the chickpea reference genome using HISAT2 (Pertea et al., 2016), a splice-sensitive aligner. To guide the alignment, splice-site and exon information were extracted from the genome annotation file (GFF format) using the Python scripts provided with the HISAT2 package. Subsequently, Gene-level read counts were generated with HTSeq (Srinivasan et al., 2020), which tallied aligned reads overlapping exonic regions.

#### 2.3.2 Differential gene expression analysis

Differential gene expression analysis was performed using the DESeq2 package (v1.38.3) in R. Before analysis, the raw count matrix was pre-filtered to retain only genes with a mean count >10 across all samples. This filter removes genes with consistently low expression that are statistically underpowered to detect differential expression and can increase the burden of multiple tests. The experimental design was modeled using the formula ∼ Group, where Group represents the experimental condition (e.g., stress treatment vs. control). The control condition was set as the reference level. Gene-wise dispersion estimates were fitted using the local fit option (fitType = ”local”) to accommodate potential mean-dispersion trends in the data.

To control for multiple hypothesis testing, the Benjamini–Hochberg procedure was applied to calculate adjusted p -values (false discovery rate, FDR). Genes with an FDR <0.01 were considered statistically significant. The FDR threshold of 0.01 was selected to ensure high confidence in the reported differentially expressed genes (DEGs), balancing stringent control of type I errors while retaining biological sensitivity. For downstream visualization, read counts were normalized and variance-stabilized using the regularized log transformation (rlog). A heatmap of the significant DEGs (ranked by FDR) was generated using the pheatmap package, with row-wise z-score scaling and clustering based on Pearson correlation distance with complete linkage Anders and Huber (2010), Rocke et al. (2015).

#### 2.3.3 Gene ontology and protein interaction predictions

The prediction of protein-protein interactions was performed using the STRING database (Search Tool for the Retrieval of Interacting Genes; https://string-db.org), with *Arabidopsis thaliana* serving as an annotation model. The generated PPI network was visualized using Cytoscape software (Kohl et al., 2011), where nodes represent proteins and edges represent interactions. Gene Set Enrichment (GSE) is the optimal approach for understanding the intrinsic biological functions associated with various genes or proteins. It simplifies the intricacy of molecular data and enhances the interpretability of biological insights (Bu et al., 2021). Functional enrichment analyses for Gene Ontology (GO) and Kyoto Encyclopedia of Genes and Genomes (KEGG) pathways were conducted using the STRING database for the candidate genes.

### 2.4 Genomic data analysis

Genome-wide association analysis was performed using the vcf2gwas software (Vogt et al., 2022), which provides an automated and reproducible framework for conducting GWAS from variant call format (VCF) files. The filtered SNP dataset (92,821 SNPs with minor allele frequency >0.05 and missing rate <0.1) was used as input along with phenotypic trait data.

The association analysis was conducted using the mixed linear model (MLM) implemented in vcf2gwas, which interfaces with the GEMMA software to fit the Q + K model (Wang et al., 2016). Population stratification was controlled by incorporating principal components (PCs) as fixed-effect covariates in the association model. The first two PCs, which capture the majority of genetic variance, were computed directly from the genotype data using PLINK’s PCA functionality implemented in vcf2gwas and included as covariates in the linear mixed model. The genetic relationship matrix (K) was constructed using genome-wide SNP markers to estimate pairwise relatedness between individuals. This kinship matrix accounts for background polygenic effects and familial relatedness, thereby reducing false-positive associations caused by cryptic relatedness within the population (Wen et al., 2018). Association tests were performed for each trait using the MLM implemented in vcf2gwas using the following command: vcf2gwas -v $vcfFile -pf $phenData -ap -lmm -cf ”PCA” -ac -o $outFolder. A significance threshold of p<0.001 was applied to identify putative marker–trait associations, balancing stringency with the ability to detect moderate-effect loci suitable for downstream integrative analyses. Subsequently, significant associations were used in conjunction with transcriptomic data to support candidate gene identification.

### 2.5 R package architecture and functional overview

**FIGURE 2:**
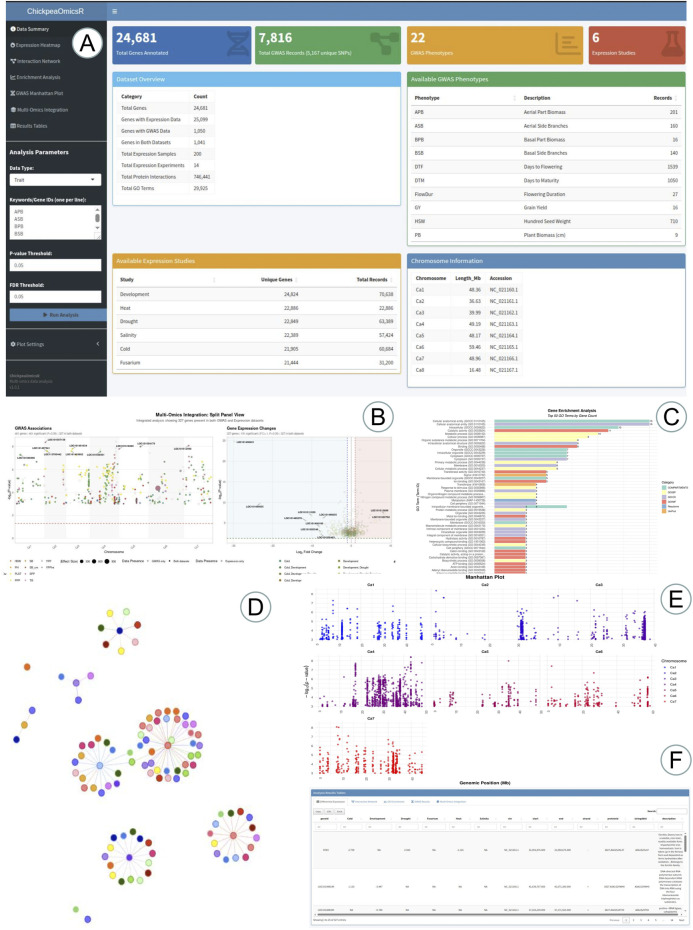
Overview of the ChickpeaOmicsR Shiny web application and its multi-omics analysis tools. **(A)** An interactive data summary dashboard providing an overview of the integrated chickpea datasets and user-defined analysis parameters. **(B)** Multi-omics integration panel combining GWAS associations with gene expression changes, enabling simultaneous visualization of genomic signals and transcriptomic responses across traits and conditions. **(C)** Gene Ontology (GO) enrichment analysis displayed as an interactive bar plot, highlighting significantly enriched biological processes. **(D)** Protein–protein interaction (PPI) network visualization with nodes representing genes/proteins and edges indicating interactions. **(E)** GWAS Manhattan plots showing the distribution of association signals across chickpea chromosomes. **(F)** Results table summarizing integrated multi-omics outputs.

To ensure continuous productivity and provide a reliable resource for chickpea analysis, we developed the ChickpeaOmicsR package using the R programming language, a widely recognized tool for statistical computing and bioinformatics. The R package can be downloaded from https://github.com/AlsammanAlsamman/ChickpeaOmicsR. It integrates various types of data, including gene expression counts from RNA-seq, protein sequence and interaction data, genome annotations, and genome-wide association study (GWAS) results, allowing users to perform a wide range of analyses. The ChickpeaOmicsR package is designed to streamline chickpea genomics and proteomics research by providing a collection of preloaded datasets, which facilitate various types of analysis. These datasets include genomic, transcriptomic, and protein data, ensuring researchers and breeders can access key information without the need for extensive preprocessing (Table 2). The package offers tools for gene expression analysis, protein-protein interaction network visualization, gene enrichment, and the integration of multi-omics data to associate genetic variants with phenotypic traits. The structure of the package is modular, providing preloaded datasets and functions that allow researchers to easily retrieve, filter, analyze, and visualize data. This flexible architecture supports a streamlined workflow for analyzing chickpea genetics and genomics, making it a valuable resource for breeders and researchers working on chickpea improvement (Figure 2).

**TABLE 2:** Summary of the ChickpeaOmicsR database components, describing the integrated genomics, transcriptomics, proteomics, and GWAS resources available for chickpea data analysis.

Database	Description
CaExpressionCounts	Gene expression counts derived from RNA-seq data, encompassing 28,891 genes across 197 samples. These data were collected and analyzed as discussed previously in this study
CaExpressionMetadata	Metadata associated with the gene expression studies, providing contextual information regarding sample types and experimental conditions
CaExpressionSignificance	Data on significant gene expression changes, particularly useful for identifying genes involved in stress responses and various developmental stages
CaGenomeAnnotation	Genome annotations for chickpea genes, detailing gene structures and functional elements. These annotations were generated using the reference genome (NCBI accession: ASM33114v1), as previously published (Varshney et al., 2014)
CaProteinEnrichment	Results of protein enrichment analyses for all chickpea genes
CaProteinSequenceInfo	Sequence information for chickpea proteins, facilitating functional annotation and comparative analyses
CaProteinInteractionNetwork	Data on protein-protein interaction networks, essential for understanding molecular interactions within chickpea. These data were sourced from the STRING database
CaGWAS	Genome-wide association study (GWAS) data for chickpea, linking genetic variants to phenotypic traits. The genome-wide association analysis was conducted as previously described in the methods, utilizing data published with ICARDA genotypes

### 2.6 Shinny R implementation

We developed an additional interface for the ChickpeaOmicsR package in the form of a Shiny web application, designed to enable users with limited R programming experience to explore integrative multi-omics analyses of chickpea genomic data. Users can easily download and run the application in RStudio, with multiple options for selecting specific genes, traits, and stress conditions. This application provides researchers and breeders with intuitive yet powerful tools to investigate complex biological datasets, including RNA-seq expression profiles, protein–protein interaction networks, gene ontology enrichment analyses, and genome-wide association studies (GWAS).

The application features six interactive visualization modules that enable comprehensive data exploration and analysis. Users can generate customizable gene expression heatmaps showing log2 fold changes across multiple studies and conditions, explore dynamic protein-protein interaction networks with expression overlays, visualize GWAS Manhattan plots displaying genomic associations across chromosomes, and create multi-omics integration plots that combine gene expression with GWAS results. Additional tools include interactive GO enrichment bar plots highlighting key biological processes, downloadable results tables for all analyses, and a comprehensive data summary dashboard providing dataset statistics. All visualizations are publication-ready with download capabilities in PNG and HTML formats, making ChickpeaOmicsR an invaluable platform for chickpea genomics research and prioritizing candidate markers for breeding programs.

The Shinny app can be downloaded from https://github.com/AlsammanAlsamman/ChickpeaOmicsRShinny, and can temporary accessed via https://samman.shinyapps.io/ChickpeaOmicsR/.

## 3 Results

### 3.1 Differentially expressed genes (DEGs) under various stress conditions

Investigation of DEGs under various stress conditions revealed distinct patterns of gene expression. Each stress condition—including drought, heat, Fusarium wilt, salinity, cold, and developmental stages—manifested unique and shared sets of DEGs. The examination of these gene expression profiles provided insights into the specific molecular responses triggered by each stressor. Analyzing data from 15 experiments and a total of 205 samples across various stress conditions posed significant challenges.

For drought stress (three experiments), PRJNA288321 showed 707 DEGs (497 downregulated and 210 upregulated). Additionally, PRJNA335939 showed 39 DEGs (17 downregulated and 22 upregulated), and PRJNA396819 showcased 901 DEGs, including 589 downregulated and 312 upregulated DEGs. For heat stress (PRJNA748749), 658 DEGs were identified (562 downregulated and 96 upregulated). In the Fusarium experiments, which included two distinct trials and a total of eight samples, PRJNA246444 revealed 802 DEGs, showcasing 282 downregulated and 520 upregulated DEGs. Concurrently, PRJNA891950 identified 169 DEGs, composed of 31 downregulated and 138 upregulated DEGs.

For salinity stress (three experiments), PRJNA232700 showed 20 DEGs (2 downregulated and 18 upregulated). Additionally, PRJNA288473 presented 36 DEGs, consisting of 33 downregulated and 3 upregulated DEGs. Meanwhile, PRJNA579008 showed 40 DEGs (18 downregulated and 22 upregulated). For cold stress (three experiments, 26 samples total), PRJNA232700 showed 4 DEGs (2 downregulated and 2 upregulated). PRJNA903665 exhibited 2387 DEGs, encompassing 1145 downregulated and 1242 upregulated DEGs. Additionally, PRJNA905065, 2200 DEGs were identified, comprising 1025 downregulated and 1175 upregulated DEGs. For developmental stages (three experiments), substantial variability in gene expression was observed. PRJNA316844 exhibited 7021 DEGs, including 3482 downregulated and 3539 upregulated DEGs. In addition, PRJNA316845 presented 7150 DEGs, consisting of 3538 downregulated and 3612 upregulated DEGs, and PRJNA413872 showed 54 DEGs (6 downregulated and 48 upregulated). All these details are presented in Table 3.

**TABLE 3:** Summary of differentially expressed genes (DEGs) identified across multiple stress conditions and developmental stages in chickpea. For each SRA experiment, the total number of DEGs (n) is shown, along with the number of up- and downregulated genes based on ${\text{log}}_{2}$ fold-change direction.

Stress condition	SRA accession	Total DEGs (n)	Regulation direction	Number of genes (n)
Drought	PRJNA396819	901	upregulated	312
			downregulated	589
	PRJNA335939	39	upregulated	22
			downregulated	17
	PRJNA288321	707	upregulated	210
			downregulated	497
Fusarium wilt	PRJNA246444	802	upregulated	520
			downregulated	282
	PRJNA891950	169	upregulated	138
			downregulated	31
Heat	PRJNA748749	658	upregulated	96
			downregulated	562
Cold	PRJNA905065	2200	upregulated	1175
			downregulated	1025
	PRJNA903665	2387	upregulated	1242
			downregulated	1145
	PRJNA232700	4	upregulated	2
			downregulated	2
Development Stages	PRJNA316844	7021	upregulated	3539
			downregulated	3482
	PRJNA316845	7150	upregulated	3612
			downregulated	3538
	PRJNA413872	54	upregulated	48
			downregulated	6
Salinity	PRJNA579008	40	upregulated	22
			downregulated	18
	PRJNA232700	20	upregulated	18
			downregulated	2
	PRJNA288473	36	upregulated	3
			downregulated	33

### 3.2 Gene intersection between different stresses

**FIGURE 3:**
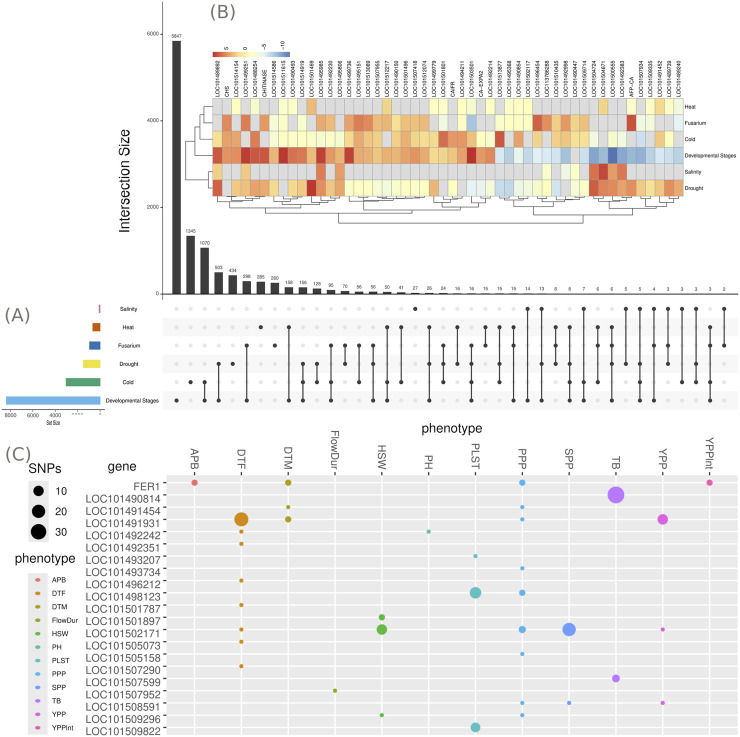
The gene intersection and multiomics analysis revealed a significant association between gene expression and various stresses and developmental stages in chickpea. **(A)** A Venn diagram illustrates shared differentially expressed genes (DEGs) across multiple chickpea studies, and **(B)** Heatmap of the top 50 genes responsive to ≥4 stresses. Rows represent stresses, columns represent genes (LOC IDs), and colors indicate $\log_{2}$ foldchange (blue: downregulated; red: upregulated). Key genes include LOC101489892 (Expansin-like B1), showing stress-context-dependent regulation: upregulated under cold, salinity, and drought stresses and during developmental stages, but downregulated under heat stress and Fusarium wilt. **(C)** GWAS analysis results, obtained using the CaGWAS database included in the ChickpeaOmicsR package, were integrated to identify the potential roles of highly significant and shared DEGs with agronomic traits, as published by (Khan et al., 2024).

We identified 2,910 DEGs common across multiple conditions. Of these, 2,380 DEGs were shared between two stress conditions, 484 were shared across three, 43 across four, and 3 across five. Intersection analysis revealed several patterns of gene sharing across different stress conditions and developmental stages in chickpea. Specifically, three DEGs were shared among heat, Fusarium wilt, cold, and developmental stages, while five DEGs were common to salinity, drought, cold, and developmental stages. Six DEGs were found to be shared among heat, Fusarium wilt, drought, and developmental stages. Furthermore, eight DEGs were shared across heat, drought, cold, and developmental stages, and sixteen DEGs were shared among Fusarium wilt, drought, cold, and developmental stages, as shown in Figure 3A. Conversely, many DEGs were condition-specific: 27 unique to salinity, 260 to Fusarium wilt, 285 to heat, 434 to drought, 1,345 to cold, and 5,847 to developmental stages, as presented in Figure 3A.

We also examined the 50 most frequently occurring DEGs (in four or more conditions) and presented the results as a heat map (Figure 3B). Our analysis revealed several notable patterns. Genes encoding Late Embryogenesis Abundant 2 (LOC101504724) and Ninja-family protein AFP2 (LOC101492383) were upregulated in salinity and drought stresses but downregulated during developmental stages (Figure 3B). The gene Expansin-like B1 (LOC101489892) was upregulated in cold, developmental stages, salinity, and drought conditions, but downregulated in Fusarium and heat stress (Figure 3B). The CHS gene, along with the genes encoding disease resistance response protein DRRG49-C (LOC101499736) and isoliquiritigenin 2′-O-methyltransferase-like (LOC101512074), was upregulated in fusarium, cold, and developmental stages but downregulated in heat, salinity, and drought stresses (Figure 3B). Genes Pathogenesis-related Protein PR-4 precursor (LOC101499251) and Thaumatin-like Protein 1b (LOC101495985) showed upregulation in Fusarium, developmental stages, salinity, and drought, while being downregulated in cold and heat conditions (Figure 3B). During developmental stages, expansin-like B1 (LOC101489892) and hexose carrier protein HEX6 (LOC101492214) were upregulated, while those from LOC101513877 to LOC101489240 were downregulated (Figure 3B). This comprehensive analysis highlights the complex and varied responses of chickpea genes to different stress conditions, providing valuable insights for future research and breeding strategies.

### 3.3 Gene enrichment analysis

Gene Ontology (GO) analysis was used to elucidate the roles of DEGs under different stress conditions, categorizing them into biological processes, cellular components, and molecular functions. By highlighting key pathways and components activated in response to stresses like drought, cold, salinity, and Fusarium wilt, GO analysis provides valuable insights. The biological annotation of the top differentially expressed genes (DEGs) under various stress conditions was carried out using the STRING online database.

**FIGURE 4:**
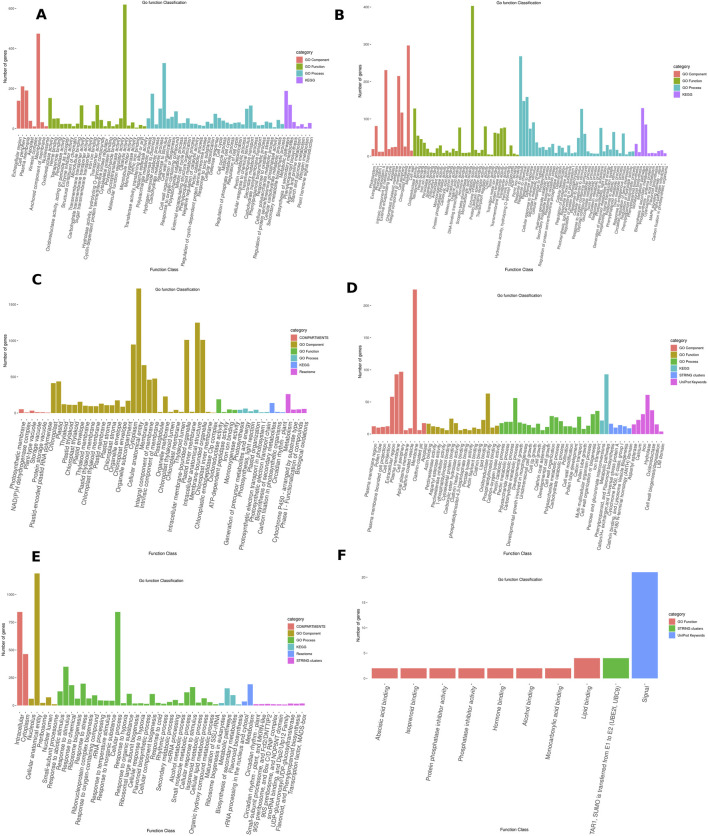
Gene enrichment analysis of significantly differentially expressed genes (DEGs) associated with various stresses and developmental stages in chickpea. **(A)** Drought stress. **(B)** Fusarium wilt. **(C)** Developmental stage. **(D)** Heat stress. **(E)** Cold stress. **(F)** Salinity stress.

Under drought stress, DEGs were significantly enriched in several biological processes, including hydrogen peroxide catabolic process, cell wall organization, pectin catabolic process, defense response, response to oxidative stress, cellular detoxification, response to toxic substances, and the phenylpropanoid biosynthetic process. These processes are critical for managing oxidative stress, reinforcing cell structure, and activating defense mechanisms under water deficit. Molecular functions enriched under drought conditions involved polysaccharide binding, glucosyltransferase activity, peroxidase activity, oxidoreductase activity, transporter activity, and tetrapyrrole binding, all of which play roles in modifying the cell wall, managing oxidative damage, and ensuring proper transport of molecules essential for stress adaptation. At the cellular level, the plasma membrane and apoplast are crucial components that facilitate water transport, mediate stress signaling, and maintain structural integrity, contributing to the plant’s overall resilience to drought stress (Figure 4A).

The enrichment analysis of DEGs in response to Fusarium wilt was conducted to gain a deeper understanding of the underlying processes. These DEGs were observed to be enriched in various biological aspects. In terms of Biological Process, the enrichment encompassed defense responses, response to biotic stimulus, response to stress, and oxidative stress, all of which are essential for counteracting pathogens. Additionally, pathways such as toxin biosynthesis, phytoalexin metabolism, and phenylpropanoid biosynthesis contribute to the plant’s chemical defense mechanisms. The molecular functions that are highly enriched include peroxidase activity, oxidoreductase activity, chitin binding, and enzyme inhibitor activity, all of which play critical roles in mitigating oxidative damage and disrupting pathogen function. At the cellular level, critical components such as the cell wall, intrinsic membrane components, and the external encapsulating structures are highlighted for their roles in forming physical barriers and facilitating immune responses. Further analysis identified key KEGG pathways, including phenylpropanoid and flavonoid biosynthesis, the Mitogen-Activated Protein Kinase (MAPK) signaling pathway, and plant-pathogen interactions, which underscore the complex regulatory networks plants activate to defend against Fusarium wilt (Figure 4B).

For the developmental stages, the DEGs were significantly enriched in various Gene Ontology (GO) categories. In terms of biological processes, key pathways were identified, including photosynthesis, light reactions, generation of precursor metabolites and energy, plastid organization, and photosynthetic electron transport in photosystem I. At the molecular level, functions related to oxidoreductase activity, ATP-dependent peptidase activity, iron ion binding, and monooxygenase activity were enriched. The cellular components involved in these developmental stages include plastoglobules, chloroplasts, the thylakoid membrane, organelle subcompartments, intrinsic components of membranes, and membrane-bounded organelles. These pathways highlight the complex molecular and cellular architecture essential for plant development (Figure 4C).

This analysis provides valuable insights into the molecular mechanisms underlying the plant’s adaptive response to heat stress. Among the Biological Processes affected, pathways such as Carbohydrate metabolic process, Clathrin coat assembly, Developmental cell growth, Cell wall organization, Polysaccharide metabolic process, and Pollen tube development were highlighted, revealing significant changes in cell growth, metabolism, and structural organization during heat stress. In terms of Molecular Functions, heat stress influences key activities such as actin binding, cytoskeletal protein binding, lipid binding, oxidoreductase activity, and phosphatidylinositol binding, suggesting critical roles in maintaining cellular stability, redox balance, and lipid signaling during stress. In addition, key cell components include the plasma membrane, the pollen tube, the extracellular region, and the cell periphery, highlighting changes at the surface of the cell and within structures vital for reproduction and communication under heat-induced conditions (Figure 4D).

For cold stress, DEGs were enriched in biological processes such as response to abiotic stimulus, the response to stress, and specifically the response to cold, indicating the activation of general and cold-specific stress responses. Additionally, processes such as Ribosome biogenesis, Ribosomal large subunit biogenesis, Response to hypoxia, Flavonoid biosynthetic process, and Rhythmic process. In terms of Cellular Components, the enrichment analysis identified significant involvement of the Cellular anatomical entity, Preribosome, Nuclear lumen, and Small-subunit processome, during cold stress. From the KEGG pathways, key processes like Ribosome biogenesis in eukaryotes underline the importance of sustaining protein synthesis under cold conditions. The Circadian rhythm - plant pathway emphasizes the role of temporal regulation in optimizing stress responses. Biosynthesis of secondary metabolites and Flavonoid biosynthesis point to the crucial role of these metabolic processes in protecting cells from cold-induced damage, particularly through the production of antioxidant compounds like flavonoids (Figure 4E).

For salinity stress, DEGs were enriched in several biological processes, highlighting the plant’s adaptive mechanisms to high salt conditions. These included abscisic acid binding, which is a critical pathway, reflecting the role of abscisic acid (ABA) in managing osmotic stress and regulating water balance in plants (Roychoudhury and Paul, 2012; Roychoudhury et al., 2013). Additionally, pathways such as Hormone binding, Isoprenoid binding, Lipid binding, Phosphatase inhibitor activity, and Alcohol binding suggest their potential roles in detoxification and the management of oxidative stress under saline conditions (Figure 4F).

### 3.4 Protein-protein interaction (PPI) network construction

**FIGURE 5:**
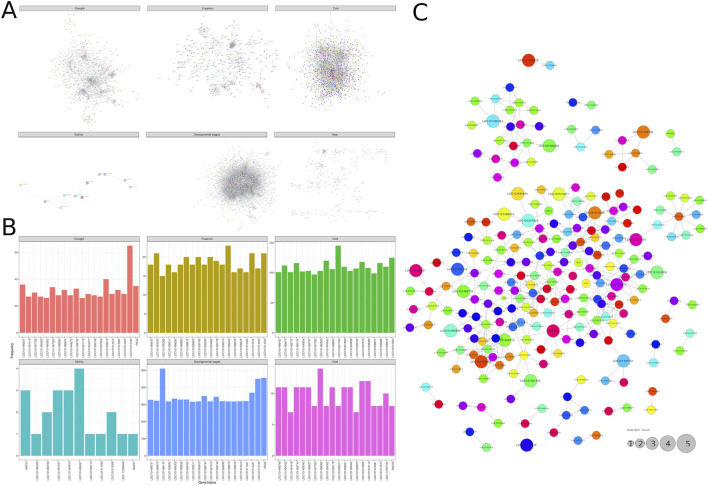
Protein-Protein Interaction (PPI) Network: **(A)** PPI network of the top differentially expressed genes (DEGs) under various stress conditions, **(B)** Bar plot showing the highly interacting proteins under different stress conditions, **(C)** PPI network highlighting key genes that are significantly frequent across five distinct stress conditions.

Plants are exposed to various stresses that significantly influence their growth and function. To understand these interactions, we conducted a thorough investigation to identify the top interacting genes associated with different stress conditions. We focused on genes involved in multiple processes crucial for managing these conditions. The protein-protein interaction (PPI) network of the top differentially expressed genes (DEGs) under various stress conditions was constructed using the STRING online database, as shown in Figure 5A.

The network highlights significant interactions between several DEGs. Notably, three genes (Bifunctional dihydrofolate reductase-thymidylate synthase (LOC101514182), LOC101509479, and Fasciclin-like arabinogalactan protein 2 (LOC101490554)) exhibited high interaction rates under drought stress (Figure 5B). Genes Photosystem I reaction center subunit psaK (LOC101506632), Chlorophyll a-b binding protein (LOC101494343), Isoliquiritigenin 2′-O-methyltransferase-like (LOC101514542 and LOC101512074) showed high interaction in response to Fusarium wilt (Figure 5B). For cold stress, genes ubiquitin-60S ribosomal protein (LOC101501706), Probable nucleolar protein 5–1 (LOC101515595), and 40S ribosomal protein (LOC101493843) had particularly high interaction rates (Figure 5B). Under salinity stress, genes Abscisic acid receptor PYL4-like (LOC101499097), ARF23, NAC domain-containing protein 7 (LOC101491337), and Protein EARLY-RESPONSIVE TO DEHYDRATION 7 (LOC101495575) had high interaction rates (Figure 5B). During developmental stages, genes Ubiquitin-60S ribosomal protein L40 (LOC101490142), UBQ5, bifunctional dihydrofolate reductase-thymidylate synthase (LOC101514182), 40S ribosomal protein (LOC101513421), glycerol-3-phosphate acyltransferase 2 (LOC101498386), NAC domain-containing protein (LOC101512115), and abscisic acid receptor PYL2 (LOC101510806) were significantly interactive (Figure 5B). Additionally, heat stress revealed high interaction rates for genes glycerol-3-phosphate acyltransferase 2 (LOC101498386), NAC domain-containing protein (LOC101512115), and abscisic acid receptor PYL2 (LOC101510806) (Figure 5B). This comprehensive analysis sheds light on the molecular mechanisms plants use to cope with various stress conditions, highlighting key genes and their interactions.

We also constructed a protein-protein interaction (PPI) network for the most frequently occurring genes under six different stress conditions. The PPI network consisted of 500 nodes and 383 edges. In this network, we examined how proteins interact, finding that each protein typically connects with about one other protein, with an average node degree of 1.53. Additionally, the network shows a moderate level of clustering, where proteins form groups of interconnected interactions, with an average local clustering coefficient of 0.278. Following a thorough analysis, we identified LOC101503501 (Protein: Monothiol glutaredoxin-S9), LOC101490854 (Protein: SRG1), and LOC101495985 (Protein: Thaumatin-like protein 1b) as key genes significantly frequent under five different stress conditions, as presented in Figure 5C. A key feature of attempted pathogen infection is the rapid production of small, redox-active molecules, such as nitric oxide (NO) and reactive oxygen species (ROS). Nitric oxide, in particular, regulates a wide range of immune responses in plants, most notably by activating a set of defense-related genes. Previous data imply that SRG1 is a central target of NO bioactivity during plant immunity, modulating the transcription of plant defense genes in response to changes in (S)NO concentrations (Cui et al., 2021; Cui et al., 2018). Thaumatin-like proteins (TLPs) are a highly complex protein family involved in host defense and developmental processes across plants, animals, and fungi. In angiosperms, they are notably diverse and classified under the PR-5 (Pathogenesis-Related-5) protein family. TLPs in plants have various biological functions, including host-pathogen interactions, stress tolerance, and cell signaling transduction. They play significant roles in responding to both biotic and abiotic stresses, such as drought, freezing, and salinity (Feng et al., 2024; Sharma et al., 2022; de Jesús-Pires et al., 2020).

### 3.5 ChickpeaOmicsR content and functionality

ChickpeaOmicsR includes multiple curated datasets, each representing essential aspects of chickpea genomics. These datasets offer users access to gene expression counts for 28,891 genes across 205 samples, genome annotation details, protein enrichment data, and protein-protein interaction networks (Table 2).

Additional datasets provide significant gene expression changes and GWAS data, which users can utilize directly within the package. Each dataset is designed to facilitate visualization and integration, allowing for streamlined workflows in chickpea research. These resources provide breeders with an in-depth view of genotype-phenotype interactions, helping to guide trait-focused breeding strategies (Figure 2).

Among its core functionalities, ChickpeaOmicsR provides tools for advanced gene expression analysis, enabling users to filter and visualize gene expression data by Gene Ontology (GO) terms, trait-related keywords, or significance thresholds. For example, breeders can examine expression heatmaps for genes related to traits such as flowering or drought tolerance. The package also supports comprehensive protein interaction network analysis, where users can visualize networks and overlay gene expression data to explore molecular interactions that underpin important traits. Additionally, ChickpeaOmicsR enables the integration of gene expression and GWAS data, helping breeders associate specific genes with phenotypic traits and providing insights that facilitate marker discovery and genetic selection.

This package demonstrates its utility in identifying flowering-related genes in chickpea through an intuitive and streamlined workflow. By using the command ”get_geneids_bygotermkeywords (c (”Flowering”))”, users can retrieve gene IDs associated with various gene ontology (GO) terms and protein families related to the flowering process, allowing quick access to specific gene clusters. The results include detailed gene sets, such as ”CL:24385”, ”GO:0048578”, and ”IPR039274”, each listing genes related to different aspects of flowering regulation. Further analysis of these genes’ association with flowering traits can be conducted using the ”get_gwas_data ()” function, which provides genome-wide association study (GWAS) data for the genes, filtered by a specified p-value threshold. This approach facilitates the identification of candidate genes linked to agronomically significant flowering traits, like days to flowering (DTF), under various environmental conditions. Additionally, the ”gene_pheno_GWASmatrix_byGeneID ()” function enables visualizing the phenotypic relevance of each gene, constructing a gene-phenotype matrix that highlights specific associations with traits such as flowering duration (FlowDur) and yield-related parameters (Figure 3C). By leveraging these functionalities, the package allows researchers and breeders to efficiently pinpoint and analyze important pathway-related genes in chickpea, supporting the development of breeding strategies aimed at optimizing and improving yield resilience.

## 4 Discussion

Chickpea is a crucial crop for alleviating protein deficiencies in developing countries, serving as a primary source of both protein and energy (Tassoni et al., 2020). However, its production faces significant challenges due to various biotic and abiotic stresses. Recent advances in high-throughput sequencing, particularly RNA-seq and whole-genome sequencing, have facilitated in-depth studies of gene expression and stress responses. This study examined gene expression in chickpea under six distinct stress conditions, providing a valuable genomic resource to support chickpea breeding efforts.

Although general-use multi-omics integration tools like mixOmics (Rohart et al., 2017), pathwayMultiomics (Odom et al., 2021), and compcodeR (Soneson, 2014) offer robust frameworks for multivariate analysis, feature selection, pathway-based integration, and testing differential expression methods, ChickpeaOmicsR distinguishes itself as the first R package specifically designed for chickpea research. In contrast to mixOmics, which is proficient in integrating N- and P-data across varied omics datasets (such as transcriptomics, proteomics, and metabolomics) but needs considerable user input and lacks curated resources specific to crops, ChickpeaOmicsR providess integrated, comprehensive workflows with pre-existing chickpea datasets (RNA-seq counts for 28,891 genes from 205 samples, standardized annotations based on the latest reference genome, GWAS findings from ICARDA accessions, and STRING-derived PPI networks), thus removing the need for manual curation and allowing breeders with minimal bioinformatics knowledge to efficiently conduct DEG identification, GO enrichment, PPI visualization, and trait-gene associations in just minutes. Unlike pathwayMultiomics, which emphasizes pathway-level integration (such as MiniMax statistics for categorical/continuous/survival outcomes) without the need for species-specific preprocessing or breeding-related functions, ChickpeaOmicsR combines transcriptomic stress responses (covering six conditions and developmental phases), GWAS signals, and PPI networks into a coherent, reproducible framework tailored for chickpea agronomic traits (like flowering time and drought tolerance). Ultimately, in contrast to compcodeR, which is mainly focused on benchmarking RNA-seq DE methods via simulation and performance comparison instead of direct biological analysis, ChickpeaOmicsR emphasizes practical use by automating species-specific pipelines and offering an easy-to-use Shiny interface for interactive exploration. Consequently, although these established instruments address general or methodological requirements, ChickpeaOmicsR addresses a vital need in chickpea genomics by providing crop-specific, breeding-ready integration that speeds up the development of stress-resilient varieties.

The analysis of differentially expressed genes revealed distinct and condition-specific gene expression patterns. Each stressor elicited unique molecular responses. Notably, drought stress experiments identified substantial numbers of DEGs (e.g., 707 in PRJNA288321, 39 in PRJNA335939, and 901 in PRJNA396819). Heat stress experiments analysis, represented by PRJNA748749 identified 658 DEGs, emphasizing the plant’s adaptive responses to high temperatures. Similarly, Fusarium infection experiments, particularly PRJNA246444 and PRJNA891950, revealed 802 and 169 DEGs, respectively, indicating critical genetic pathways involved in pathogen resistance. These insights are crucial for breeding Fusarium-resistant chickpea varieties. Salinity stress, analyzed through three experiments, showed limited changes in gene expression in chickpea. Cold stress responses revealed over 2,000 DEGs, revealing complex regulatory mechanisms enabling chickpea to withstand low temperatures. Developmental stages exhibited the highest variability in gene expression, with their datasets identifying over 7,000 DEGs. This substantial variability underscores the dynamic nature of gene expression throughout chickpea’s growth and development phases. Overall, these findings provide a detailed understanding of the genetic basis of stress responses in chickpea.

Under drought stress, DEGs were significantly enriched in categories such as the cell wall organization, defense response, and response to oxidative stress. Cell wall encompasses a series of biochemical processes responsible for the synthesis, modification, and maintenance of the cell wall in plants. The cell wall provides mechanical support, responding to environmental factors such as pathogens, drought, and other stresses, and helps regulate water balance (Minic and Jouanin, 2006; Moore et al., 2008; Tenhaken, 2015).

For Fusarium wilt, the enrichment of DEGs in response to biotic stimuli, flavonoid biosynthesis, the MAPK signaling pathway, and plant-pathogen interactions emphasizes the importance of innate immunity and secondary metabolite production in pathogen resistance. MAPKs play a key role in signaling multiple defense responses, including the biosynthesis and signaling of plant stress and defense hormones, the generation of reactive oxygen species (ROS), stomatal closure, activation of defense genes, phytoalexin biosynthesis, cell wall strengthening, and hypersensitive response (HR) cell death (Meng and Zhang, 2013). Developmental stages showed enrichment in photosynthesis and the thylakoid membrane, highlighting their roles in plant growth and stress responses. Photosynthesis, the key bioenergetic process, occurs in the chloroplast. The components of the photosynthetic machinery are embedded in a highly dynamic matrix, the thylakoid membrane. This membrane can adapt during developmental transitions and under stress conditions. Thylakoid lipids, such as prenyllipids and carotenoids, play essential roles in processes like electron transport and photoprotection (Rottet et al., 2015; Vothknecht and Westhoff, 2001).

For heat stress, the significant enrichment of DEGs in actin binding, cytoskeletal protein binding, and oxidoreductase activity highlights crucial role in the plant response to heat stress by maintaining cell structure and facilitating intracellular transport. For cold stress, DEGs were enriched in response to cold, circadian rhythm (plant), and response to abiotic stimulus. Circadian rhythms, the internal biologiregulating various plant functions, such asesponses to cold stress. Circadian rhythms are important for controlling different plant functions, like leaf and organ movements, stomatal opening, growth, and signaling. The C repeat binding factor (CBF) genes, which help plants tolerate cold, are regulated by key clock proteins, CCA1 (Circadian Clock Associated 1) and LHY (Late Elongated Hypocotyl) (Webb, 2003; Maibam et al., 2013). Salinity stress revealed enrichments in abscisic acid binding, hormone binding, isoprenoid binding, and phosphatase inhibitor activity, indicating biochemical strategies for coping with high salt levels. Abscisic acid (ABA) binding plays a crucial role in how plants respond to various stresses, especially salinity stress, which can adversely affect growth and development. ABA acts as a signaling molecule that helps regulate various physiological processes, including managing osmotic stress and regulating water balance in plants (Roychoudhury and Paul, 2012; Roychoudhury et al., 2013).

Our investigation of the protein-protein interaction (PPI) network, constructed from differentially expressed genes (DEGs) shared across stress conditions, revealed a connected module central to chickpea’s generalized stress response. The network (Figure 5C), comprising 500 nodes and 383 edges, was not random; key hubs such as LOC101503501 (Monothiol glutaredoxin-S9), LOC101490854 (SRG1), and LOC101495985 (Thaumatin-like protein 1b) exhibited significantly higher connectivity.

The centrality of these hubs is functionally critical. LOC101503501 (monothiol glutaredoxin-S9), acts as a central redox sensor and transducer. Its high connectivity suggests it integrates signals from various stress-induced redox changes, coordinating the activity of downstream transcription factors and enzymes to reestablish cellular homeostasis (Zaffagnini et al., 2012; Chai and Mieyal, 2023; Ogata et al., 2021). Similarly, LOC101490854 (SRG1), a regulator of defense genes, is positioned to amplify and distribute defense signals initiated by different stressors via nitric oxide (NO) signaling. Its hub status implies it functions as a regulatory bottleneck, making its expression crucial for an effective immune response (Cui et al., 2021; Cui et al., 2018). LOC101495985 (Thaumatin-like protein) likely serves as a convergence point for pathogen defense and abiotic stress signaling, potentially through interactions with cell wall modifiers or membrane receptors (Feng et al., 2024; Sharma et al., 2022; de Jesús-Pires et al., 2020). Therefore, these hubs are not only differentially expressed but also serve as functional integrators. Their disturbance during stress and their high connectivity allow them to impact the network state significantly, making them strong candidates for improving multi-stress tolerance in chickpea.

The gene intersections among different stress conditions in chickpea have provided significant insights into the plant’s adaptive mechanisms. By examining the top differentially expressed genes (DEGs) shared across six distinct stress conditions, we identified key patterns of gene regulation that highlight the complexity of chickpea’s stress responses. For instance, LOC101489892 (expansin-like B1) was upregulated in cold, developmental stages, salinity, and drought but downregulated in Fusarium wilt and heat stress. Similarly, the CHS gene was upregulated in the wilting, cold, and developmental stages of Fusarium wilt, but downregulated in heat, salinity, and drought conditions, indicating its pivotal roles in managing various stresses. Additionally, genes specific to each stress condition suggest specialized pathways that chickpea employs to cope with individual stress types. The identification of these stress-responsive genes, especially those frequently occurring under multiple stress conditions, provides valuable targets for future research aimed at enhancing stress tolerance through genetic and breeding strategies.

To disseminate the transcriptomic results of this study and provide more adaptive resources for chickpea breeding, we developed an R package called ”ChickpeaOmicsR”. ChickpeaOmicsR is an innovative R package designed to streamline multi-omics data analysis specifically for chickpea research, addressing the growing need for accessible, integrative bioinformatics tools in agricultural genomics. As a comprehensive toolkit, ChickpeaOmicsR supports chickpea breeders and researchers by providing specialized functions for gene expression analysis, protein interaction network visualization, and genome-wide association studies (GWAS). This package enables researchers to explore and analyze key biological pathways and traits, such as flowering, salinity tolerance, and stress adaptation, critical to advancing chickpea breeding programs and improving crop resilience. ChickpeaOmicsR can be used to make data-driven decisions by providing tools to identify critical genes associated with essential breeding traits, such as stress tolerance, disease resistance, and flowering time. It allows users to easily locate these genes and visualize complex interactions by analyzing protein-protein interaction networks, overlaying expression data to gain deeper insights into gene interactions relevant to these critical traits. Additionally, ChickpeaOmicsR facilitates the integration of multi-omics data, enabling users to combine gene expression and GWAS data to uncover connections between genetic variation and phenotypic traits, thus prioritizing candidate markers for marker-assisted selection. With preloaded datasets and gene enrichment tools, the package also grants access to annotated genomic data, offering valuable insights into pathways and biological processes vital to chickpea breeding and supporting targeted breeding objectives. Additional insights can be generated using this package, which supports gene annotation, selection, fine-mapping, protein network analysis, and experimental validation. One key advantage is that all this data is interconnected, allowing users to seamlessly link information across different analyses and explore it in both directions.

Our pipeline was previously applied during its development phase in the published study by (Awaly et al., 2025). In that work, the authors successfully investigated multi-omics interactions between gene expression and genome-wide association analyses to identify candidate targets for salinity tolerance in chickpea. The pipeline was used to pinpoint target genes for real-time PCR validation and provided a coherent framework for understanding their involvement in chickpea resilience and responses to salinity stress. In addition, ChickpeaOmicsR generated functional annotation profiles indicating that these genes harbor genomic variants with significant associations to key agronomic traits, including days to maturity, plant height, and seed weight. Notably, 13 differentially expressed genes (DEGs) were specifically linked to yield- and flowering-related traits, and their expression patterns under salinity stress were subsequently validated using real-time PCR. This example clearly demonstrates that the use of ChickpeaOmicsR in chickpea research can provide deeper biological insight and pave the way for annotation-based breeding programs.

By incorporating ChickpeaOmicsR into breeding programs, chickpea researchers can better understand the molecular underpinnings of crop resilience and productivity. Each function in the package is tailored to facilitate data analysis in a manner that supports breeders’ goals, from discovering candidate genes to visualizing interaction networks. ChickpeaOmicsR exemplifies a valuable resource for the chickpea research community, promoting collaborative work, enhancing understanding of complex traits, and ultimately contributing to the advancement of chickpea breeding.

While the ChickpeaOmicsR package provides a comprehensive, integrated resource for multi-omics analysis in chickpea, it is important to acknowledge its current limitations. A primary challenge in plant genomic database development, unlike the more standardized field of human genomics, is the absence of a universally approved resource for gene nomenclature and genome assembly versions. This lack of standardization complicates the creation and, more critically, the ongoing updating of annotation databases with new information. Consequently, our package relies on hard-cored annotation datasets that users cannot dynamically update, maintain, or expand. This limitation is not inherent to the tool’s design but stems from the fundamental difficulty of connecting and harmonizing biological information when simple gene identifiers are not standardized across studies and resources. To address this in future updates, we plan to integrate BLAST-based gene identification functionality, providing a flexible ground for matching user-provided sequences to our curated identifiers. Additionally, the current version of ChickpeaOmicsR does not support sequence-based analysis (e.g., direct FASTA input or sequence alignment). We plan to add this capability and strongly encourage users to utilize standard NCBI-based gene identifiers (LOC IDs) to ensure compatibility and accuracy when conducting integrated analyses using our package’s expression, interaction, and GWAS modules.

## Data Availability

All data supporting the findings of this study are available within the article and/or its supplementary materials. ChickpeaOmicsR can be accessed https://github.com/AlsammanAlsamman/ChickpeaOmicsR. The Shinny app can be downloaded from https://github.com/AlsammanAlsamman/ChickpeaOmicsRShinny, and can temporary accessed via https://samman.shinyapps.io/ChickpeaOmicsR/. Updates or changes to the Shiny application website will be communicated via the R package webpage, and inquiries may be directed to the corresponding authors.
